# Molecular cytogenetic and phenotypic characterization of Phelan McDermid and 22q13 duplication syndrome: a case report

**DOI:** 10.1186/s13039-022-00629-7

**Published:** 2022-12-17

**Authors:** Yousif Khalifa, Hisham Y. Hassan, Anja Weise, Thomas Liehr, Haya Alkhayyat

**Affiliations:** 1 Department of Pediatrics, Bahrain Defence Force Hospital, Riffa, Kingdom of Bahrain; 2 Banoon ART and Cytogenetics Centre, Bahrain Defence Force Hospital, P.O. Box: 28743, Riffa, Kingdom of Bahrain; 3 University Hospital Jena, Friedrich, Jena, Germany

**Keywords:** Phelan-McDermid syndrome (PHMDS), Ring chromosome 22 (r(22)), 22q13 deletion, 22q13 duplication

## Abstract

**Background:**

Phelan-McDermid syndrome (PHMDS) is a rare genetic disorder mostly caused by haploinsufficincy of *SHANK3* gene, and characterized by neonatal hypotonia, developmental delay, minor dysmorphic features, seizures and behavior problems. Literature of this syndrome is scanty and confusing, and represents a challenge for pediatricians, in terms of finding the correct diagnoses.

**Case presentation:**

In a postnatal case with hypotonia and dysmorphic features a de novo ring chromosome r(22) leading to in parallel microdeletion and micro duplication in 22q13 was diagnosed by banding cytogenetics, and further characterized in detail by molecular cytogenetic and chromosomal microarray.

**Conclusion:**

Here a rare PHMDS case caused by a r(22) is presented. Less than 10 comparable cases are reported in the literature. The present case highlights the importance of conducting genetic counseling and appropriate genetic tests for newborns with mild dysmorphic features.

## Background

Ring chromosome 22/r(22) syndrome [ORPHA: 1446] is a rare chromosomal aberration with a prevalence of 1/1,000,000 and usually a de novo event; however, there are some familial cases reported. Especially familial r(22) cases may be without phenotypic effects, as in those no crucial genetic material was lost or gained. Clinical manifestations in deleterious r(22) cases may include developmental and speech delay, global intellectual disability, growth retardation, microcephaly, hypotonia and mild to prominent facial dysmorphic features, such as large ears and bulbous nose [1–4]. Psychological and behavioral problems including aggressive behavior, as well as hyperactivity, bipolar affective disorder, self-injurious behavior, and autistic spectrum disorders are relatively common among patients with this chromosomal abnormality [5–8]. Seizures of different types including febrile, generalized tonic–clonic, focal, absence seizures and peculiar features can be present in about 25% of the cases with 22q13 deletion [9, 10].

Skin disorders and pigmentation abnormalities were observed in a few number of cases with constitutional mosaicism of r[22] chromosome, such as hypomelanosis, hypochromic maculae and patchy depigmentation in different part of the body [11, 12].

Speech delay is one of the common features among individuals with 22q13 deletion, this region could be a candidate genomic segment to carry genes involved in speech development and autistic spectrum in childhood [13].

Besides copy number variants (CNVs) due to ring chromosome formation, a common mechanism how ring chromosomes may lead to clinical problems is, that they tend to lead to be lost or duplicated as a whole in part of the patient’s cells. Thus, mosaics like mos 46,XX/46,XX,r(22)/45,XX, − 21/47,XX,r(22),+r(22) can be observed. According to the mosaic status in different tissues, various phenotypic deviations may be observed in basically similar r(22) cases [14]. According to [15] r(22) has been seen to be associated with PHMDS in at least 5 cases, yet. Here we report another such case, which was not suspected to be PHMDS, before a r(22) was observed in banding cytogenetics.

## Case presentation

**Fig. 1:**
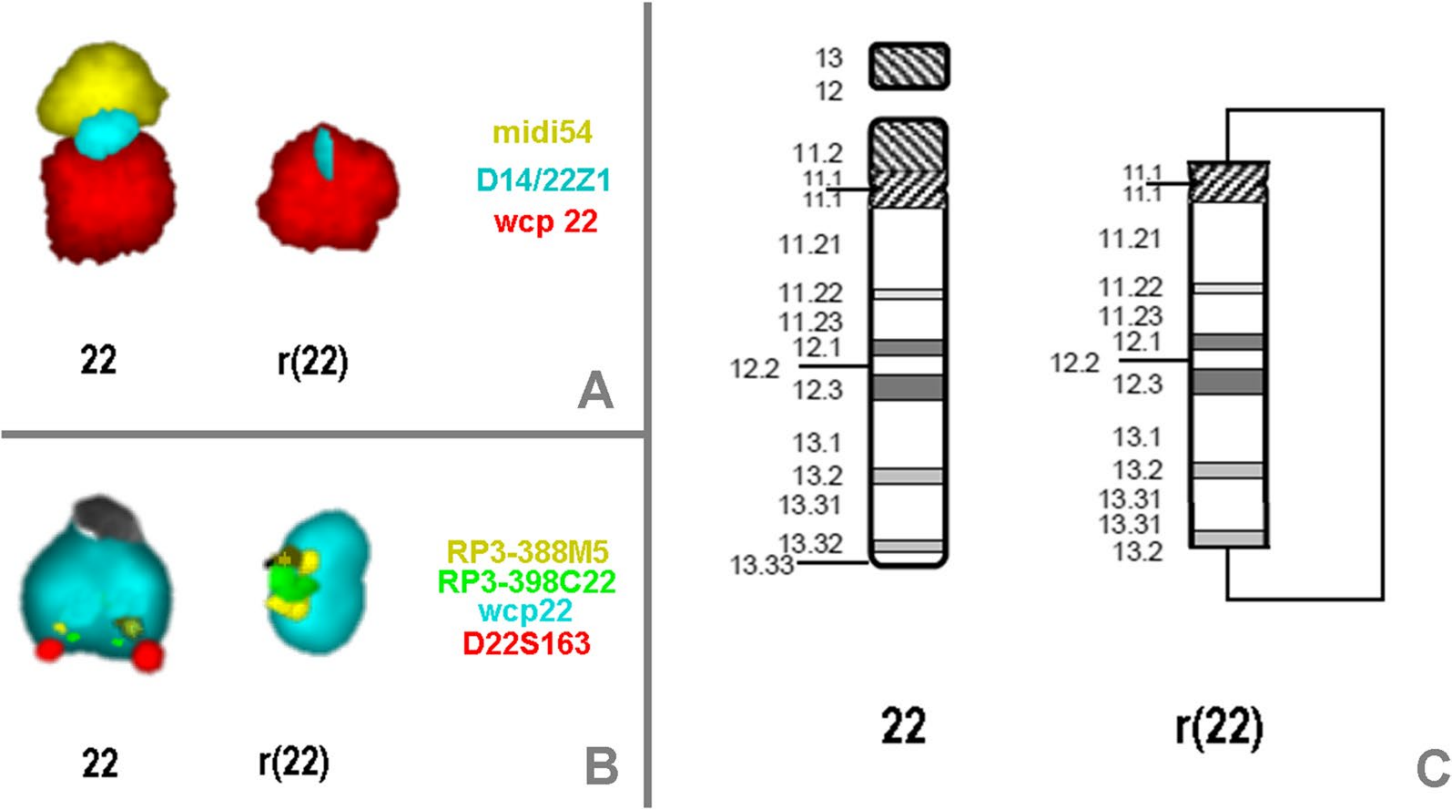
FISH using the probes as shown in (**a**), and (**b**) led to the characterization of the r(22) as a r(22)(::p11.1- > q13.31::q13.31- > q13.2::)—scheme shown in (**c**)

A full-term female infant was born by lower segment caesarian section after breech presentation. Clinical examination indicated mild neonatal hypotonia, feeding difficulties, mild facial dysmorphism expressed as small rounded face and microcephaly (head circumference at birth was 33 cm), synophrys, and a birth weight of 3.0 kg. After one month, she was admitted to the general pediatric ward with short history of cough, fever, cyanosis and change of voice particularly during crying. Otherwise, she was active, with normal heart rate and blood pressure, and no seizure or abnormal movements. Her weight was 2.66 kg, with head circumference of 34 cm being below 3rd percentile. Respiratory examination showed mild stridor, otherwise, auscultation of the chest revealed equal bilateral breath sounds with scattered rhonchi. Respiratory panel indicated human rhinovirus. Her cardiovascular and central nervous systems were unremarkable, abdomen was soft with no distension, palpable masses or organomegaly. She was treated symptomatically as a case of bronchiolitis and at the same time metabolic and chromosomal analysis were requested to investigate the causes of her failure to thrive. Karyotype revealed heterozygous ring chromosome 22 with a karyotype of 46,XX,r(22) (Fig. 1). The patient was discharged home in a stable condition after she started to gain weight in the hospital, and a clinical dietician was involved in treatment protocol. Two weeks later, she was readmitted to the pediatric ward with history of cough, fever and shortness of breath. At examination she showed mild respiratory distress as intercostal and subcostal retraction, and X-ray evidenced of bronchopneumia. Fiber optic study confirmed diagnosis of laryngomalacia.

Subsequent single nucleotide polymorphism (SNP)-array analysis (HumanCytoSNP-12v2.1, Illumina) identified two adjacent CNVs in chromosome 22:

- an interstitial duplication of the long arm of chromosome 22 (arr[GRCh37] 22q13.2q13.31(43682699_46704243) × 3) of approximately three megabases (Mb). This region is not polymorphic (DGV, gnomAD) and includes 24 OMIM genes (3 of them are classified as disease causing: *ATXN10, FBLN1* and *PPARA*; see Table 1). Duplications 22q13.2q13.31 were not described in the Achropuce, Decipher, Clingen and Pubmed databases. Thus, this copy number variant (CNV) is of uncertain significance.
- The adjacent distal heterozygous deletion of the long arm of chromosome 22 (arr[GRCh37] 22q13.31q13.33(46722256_51169045) × 1) was approximately 4.4 Mb in size. This region is not polymorphic (DGV, gnomAD) and includes 37 OMIM genes (12 of them are classified as disease causing including: *TRMU, CELSR1, ALG12, MLC1, MOV10L1, TUBGCP6, SCO2, TYMP, CHKB, SBF1*, *ARSA, and SHANK3;* see Table 2) [16–18]. Deletion of *SHANK3* and the terminal region of the long arm of chromosome 22 are responsible for autosomal dominant inherited PHMDS [OMIM #606,232, ORPHA: 48652] [19–21]. This deletion is pathogenic due to haploinsufficiency of the included autosomal dominant genes or damasking recessive mutations on the second allele and contributes to the patient’s phenotype.

**Table 1:** OMIM Morbid annotated genes with gene coordinates, mode of inheritance (AD: autosomal dominant) and associated clinical phenotypes for arr[GRch37] 22q13.2q13.31(43682699_46704243) × 3

Gene	Disease, # OMIM entry	Gene coordinate Chr 22 (GRCh38)	Mode of inheritance	Phenotype features
*FBLN1* Fibulin 1	Synpolydactyly-2 (SPD2) #608,180	45,502,883–45,601,135	AD	Hand syndactyly/synpolydactyly (3rd or 4th digits), feet Syndactyly (2nd, 3rd, 4th digits), hand and feet metatarsal synostosis (3rd and 4th digits), hand and feet Symmetric, bilateral malformations
*ATXN10* Ataxin 10	Spinocerebellar ataxia-10 (SCA10) #603,516	45,671,834–45,845,307	AD repeat expansion (ATTCT)n	Progressive cerebellar ataxia and atrophy, scanning speech, seizures, hyperreflexia, cognitive impairment, dementia, nystagmus, dysphagia, urinary incontinence, age at onset 14 to 44 years
*PPARA* Peroxisome proliferator-activated receptor-alpha	Susceptibility to hyper-apobetalipoproteinemia	46,150,526–46,243,756	-	Physiological function during various nutritional states, possible role in several chronic diseases (PMID: 10,839,530)

**Table 2:** OMIM Morbid annotated genes with gene coordinates, mode of inheritance (AD: autosomal dominant, AR: autosomal recessive, Mi: mitochondrial), and associated clinical phenotypes for arr[GRCh37] 22q13.31q13.33(46722256_51169045) × 1

Gene	Disease, # OMIM entry	Gene coordinates Chr 22 (GRCh38/hg38)	Mode of inheritance	Phenotype features
*TRMU* tRNA 5-methylaminomethyl-2-thiouridylate methyltransferase	Transient infantile liver failure (LFIT) #613,070	46,335,714–46,357,340	AR	Acute liver failure, hepatomegaly, poor feeding, pale-gray skin, lactic acidosis, laboratory abnormalities, onset usually at 2 to 6 months of age, liver size and functions return to normal after 3 to 4 months but may have persistent hypotonia
	Aminoglycoside-induced deafness #580,000		Mi	Aminoglycoside-induced hearing loss
*CELSR1* Cadherin EGF LAG seven-pass G-type receptor 1	Lymphatic malformation-9 (LMPHM9) #604,523	46,361,174–46,537,620	AD	Lower limbs lymphedema, lymphangiectasia, lymph backflow, onset in first decade
*ALG12* ALG12 alpha-1,6-mannosyltransferase	Congenital disorder of glycosylation type Ig (CDG1G) #607,143	49,859,311–49,918,438	AR	Low birth weight, failure to thrive, progressive microcephaly, facial dysmorphism, Patent foramen ovale and ductus arteriosus, male cryptorchidism, delayed ossification, rhizomelic limb shortening, feet malformation, psychomotor retardation, frequent respiratory infections
*MLC1* Modulator of VRAC current 1	Megalencephalic leukoencephalo-pathy with subcortical cysts-1 (MLC1) #604,004	50,059,391–50,085,875	AR	Macrocephaly, ataxia, seizures, spasticity, delay in motor development, mild mental retardation, cerebral MRI findings, onset in infancy
*MOV10L1* Mov10-like 1	Spermatogenic failure-73 (SPGF73) #619,878	50,090,006–50,161,687	AR	Male infertility
*TUBGCP6* Tubulin-gamma complex-associated protein 6	Microcephaly and chorioretinopathy-1 (MCCRP1) #251,270	50,217,694–50,245,023	AR	Short stature, microcephaly, facial dysmorphism, delayed psychomotor development, mental retardation, cerebral MRI findings
*SCO2* SCO2 cytochrome c oxidase assembly protein	Mitochondrial complex IV deficiency nuclear type 2 (MC4DN2) #604,377	50,523,568–50,526,442	AR	Facial dysmorphism, hypertrophic cardiomyopathy, respiratory insufficiency, feeding difficulties, severe hypotonia, myopathy, global developmental delay, dystonia, decreased/absent reflexes, cerebral MRI findings, sensorimotor axonal or demyelinating polyneuropathy, lactic acidosis, laboratory abnormalities, onset in infancy
	Myopia-6 (MYP6) #608,908		AD	High-grade myopia
*TYMP* Thymidine phosphorylase	Mitochondrial DNA depletion syndrome-1 (MTDPS1) #603,041	50,525,752–50,530,085	AR	Progressive weight loss, sensorineural hearing loss, ptosis, gastrointestinal problems, myopathy, leukoencephalopathy, sensorimotor axonal/demyelinating progressive peripheral neuropathy, lactic acidosis, laboratory abnormalities, onset in second to fifth decade
*CHKB* Choline kinase, beta	Megaconial-type congenital muscular dystrophy (MDCMC) #602,541	50,578,963–50,582,849	AR	Microcephaly, dilated cardiomyopathy, ichthyosis, Muscle weakness/dystrophy, mental retardation, delayed motor development, poor speech development, onset at birth
*SBF1* SET-binding factor 1	Charcot-Marie-Tooth disease type 4B3 (CMT4B3) #615,284	50,445,000–50,475,035	AR	Scoliosis, progressive peripheral neuropathy causing distal limb muscle atrophy/weakness, gait abnormalities, distal sensory impairment, areflexia, onset between 5 and 20 year
*ARSA* Arylsulfatase A	Metachromatic leukodystrophy (MLD) #250,100	50,622,754–50,628,152	AR	Optic atrophy, urinary incontinence, mental deterioration, loss of speech, hypotonia, muscle weakness, seizures, ataxia, dystonia, spastic tetraplegia, progressive polyneuropathy, psychiatric manifestation
*SHANK3* SH3 and multiple ankyrin repeat domains 3	Phelan-McDermid syndrome (PHMDS) #606,232	50,672,823–50,733,212	AD	Tall stature, dolicho-/macrocephaly, facial dysmorphism, neonatal feeding difficulties and hypotonia, global/delayed developmental delay, Absent or delayed speech development, moderate to severe mental retardation, seizures, increased tolerance to pain, abnormal reflexes, autistic features, aggressive behavior
	Schizophrenia-15 (SCZD15) #613,950		AD	Schizophrenia, borderline to moderate mental retardation, hyperactivity

To confirm the SNP-array results, fluorescence in situ hybridization (FISH) studies were carried out. Centromeric probe D14/22Z1 (Zytovison, Bremerhaven, Germany), a probe for all acrocentric short arms (midi54) [22], and as locus specific probes the subtelomeric probe D22S163 (Abbott/Vysis, Wiesbaden, Germany in 22q13.33), RP3-388M5 (in 22q13.-2 ~ 13.31; chr22:44,142,192–44,319,759) and RP3-398C22 (in 22q13.31; chr22:46,075,907–46,165,234) were used to further characterize the r(22). A whole chromosome paint (wcp) for chromosome 22 was applied as control probe [23]. It could be shown that almost the whole short arm of chromosome 22 was lost on the ring chromosome and one breakpoint of r(22) is in 22p11.1, covered by probe D14/22Z1. Besides by application of the three locus specific probes it could be confirmed that the subtelomeric region was deleted in the r(22) and it was revealed that the region being duplicated according to SNP-array results is inverted duplicated (see Fig. 1). Therefore, the karyotype of the case according to ISCN 2020 nomenclature [24] is 46,XX,r(22)(::p11.1- > q13.31::q13.31- > q13.2::).ish r(22)(wcp22 + ,midi54-,D14/22Z1dim,RP3-388M5 +  + ,RP3-398C22 +  + ,D22S163-).arr[GRCh37] 22q13.2q13.31(43682699_46704243) × 3),22q13.31q13.33(46722256_51169045) × 1).

Currently, the patient is one year and eight months old, active, cheerful, playful, growing well, and still rely on commercial infant milk formula. She was born with two small hemangiomas; one of them was on the front head which gradually faded, and the other on the sacral area which is regressing in size with age; so far hemangiomas are no specific features of PHMDS and are common among newborns. Her weight is 10.9 kg on the 50th percentile, length is 80 cm on the 50th percentile, and head circumference is 44 cm on 5th percentile. She is achieving her developmental milestones as she can stand and started walking.

## Discussion and conclusion

PHMDS is characterized by wide range of phenotypic and clinical features due to the highly diverse deletion sizes and different genes involved. The case presented here has a r(22) which was present in each studied cell of the patient. There was no evidence for a mosaic karyotype as reported for other r(22) cases in the literature [12, 25]. In the 22q13.31q13.33 deletion identified in the proband there are 12 genes involved, one of the important genes in this region related to the early speech development is *SHANK3* located at 22q13.3, its expression plays a pivotal role in the development of the cerebral cortex and the cerebellum, and therefore, it is a strong candidate gene for neurobehavioral disorders [26], dysmorphic features, autism spectrum disorders, bipolar disorders, hyperactivity, gastrointestinal symptoms, retinopathy, and cardia malformations [27]. Although [28] reported that clinical features of patients without *SHANK3* deletion were similar to that of patients having the *SHANK3* deletion which may suggest that other genes within this region may play a role in the phenotypes of PHMDS.

Moreover, the *ATXN10* and *FBLN1* genes which are involved in the 22q13.2q13.31 duplication, and *CELSR1* gene encode proteins that are involved in neurodevelopmental features, and their copy number variation may contribute to the clinical phenotypes of PHMDS as well [28, 29], while the deletion of *ARSA* may cause remarkable loss of myelin in the central nervous system, resulting in different neurological problems [12]. In addition to the role of *SHANK3*, the deletion of the *ALG12, MLC1, TUBGCP6, SCO2 and CHKB* genes located in the 22q13.31q13.33 region may have contributed in the mild dysmorphic features in the proband, whereas the deletion of the *TRMU, ARSA* genes may be involved in the mild hypotonia (https://www.omim.org/). The PPARA gene which is known to play a role in lipid metabolism pathways, and contributes to the susceptibility to hyperapobetalipoproteinemia and some other chronic disease [30]. However, although this gene is duplicated in the proband but all lipids profile are within the normal ranges.

While it is still early to evaluate our case regarding possible psychological and behavioral and other chronic disorders, we may conclude that the clinical characteristics including dysmorphic features like small head, small rounded face and mild hypotonia are a consequence of her chromosomal imbalance. And the level of the mild dysmorphic features in the proband might be a result of phenotypic overlay with the adjacent duplication including the *ATXN10* duplication, which might be in turn neuroprotective and modifies the severity of PHMDS features.

In this case report we describe the molecular cytogenetic and clinical characteristics of a one year and eight months female diagnosed with PHMDS involving deletion of *SHANK3* gene, and 22q13 duplication syndrome with mild hypotonia and dysmorphic features. This case sheds lights on the importance of offering genetic counseling and genetic testing for newborns with mild dysmorphic features, which could be due to genetic factors. It is highly recommended to follow up the children in order to diagnose any possible behavioral or psychological disorders, which may appear in the future, since the patient harbors a duplication of 22q13, which may increase the risk of developmental delay as well as for learning and intellectual difficulties. Taking into account that early diagnosis of such disorders might be helpful in patient’s management and treatment.

## Data Availability

Please contact the corresponding author for data requests.
